# The Delivery Disconnect: Why We Could Not Model the Impact of Foreign Aid Reductions on Vertical HIV Transmission and What Remains Evident About Strategic Priorities

**DOI:** 10.1002/jia2.70129

**Published:** 2026-06-19

**Authors:** Alyssa Bilinski, Andrea Ciaranello, Amrutha Ramaswamy, Magdalene Walters, Karen Webb, Jeffrey W. Imai‐Eaton

**Affiliations:** ^1^ Department of Health Services, Policy, and Practice Brown University School of Public Health Providence Rhode Island USA; ^2^ Department of Biostatistics Brown University School of Public Health Providence Rhode Island USA; ^3^ Medical Practice Evaluation Center and Division of Infectious Disease Massachusetts General Hospital Boston Massachusetts USA; ^4^ MRC Centre for Global Infectious Disease Analysis School of Public Health Imperial College London London UK; ^5^ Organization for Public Health Interventions and Development (OPHID) Harare Zimbabwe; ^6^ Center for Communicable Disease Dynamics Department of Epidemiology Harvard T. H. Chan School of Public Health Boston Massachusetts USA

1

Preventing HIV acquisitions in children has been one of the most impactful achievements in human health. Over the past two decades, global HIV incidence among children has fallen about 75%, from approximately 490,000 in 2005 to 120,000 in 2024, as antiretroviral therapy (ART) coverage among pregnant women with HIV rose from 10% to 84% [1, 2].

In 2025, upheaval to US foreign assistance for health threatened this progress. Early model‐based analyses projected that interruptions of HIV services previously supported by PEPFAR could substantially increase HIV transmission and AIDS‐related deaths [3, 4, 5, 6]. These models sought to illustrate potential implications of drastic cuts, in part to support funding waivers for “life‐saving HIV services,” but were limited to simplified “what if” scenarios amidst policy uncertainty. We initially sought to conduct a model‐based analysis of the impact of discontinuing foreign assistance for reproductive, maternal, newborn, and child health (RMNCH)—beyond direct HIV‐related services—on vertical HIV transmission. However, limited data on both service interruptions and the flow of HIV versus non‐HIV investment dollars left us uncomfortable generating quantitative projections as planned.

Nevertheless, precise projections are not the only output models offer. Amidst uncertainty, modelling can identify generalizable insights: which factors most shape outcomes and which conclusions are robust across scenarios. In what follows, we first review uncertainties that prevented us from generating quantitative projections and then highlight two qualitative conclusions that hold across plausible scenarios: (1) systems investment remains critical for prevention of vertical transmission (pVT); and (2) the impact of lenacapavir‐based pre‐exposure prophylaxis (PrEP) depends on this infrastructure.

What didn't we know? We encountered two data limitations. The first was the interruption of routine reporting. PEPFAR's progress on pVT has historically been documented in national health management information systems. Similar aggregate results reported directly to PEPFAR have been publicly shared on a quarterly basis. However, in 2025, the US government stopped routine reporting, releasing only limited quarter 3 2025 data in April 2026 [7]. This rendered it challenging to understand how policy shifts affected care.

Second, we struggled to characterize the relationship between funding and services, as national governments, PEPFAR (for HIV‐related services), USAID (for myriad non‐HIV‐related services, including reproductive and maternal health), the Global Fund and other donors funded closely related services at the same delivery sites. For example, at an antenatal clinic, staff may have been paid through one mechanism, supplies purchased through another and overhead or specific services funded through a third. We could not determine who paid for what, which components were at risk of reductions under different restructuring scenarios or what would remain if the US government continued funding “life‐saving HIV services” while cutting everything else. These distinctions often did not correspond to how care was organized at the point of delivery.

What we do know, even absent these data, is that eliminating VT depends on a cascade of services that extend beyond HIV care. In 2025, the US government articulated eliminating VT as a global health priority [8], even as it discontinued RMNCH support. We believe this is shortsighted because pVT does not exist in isolation, but rather as the impact of a cascade including HIV‐specific and non‐HIV‐related care. USAID cuts and aid restructuring threaten primary and antenatal care infrastructure on which HIV testing, diagnosis and treatment initiation depend. Reductions in family planning programmes may increase unintended pregnancies [9]. These same programmes are often the entry point for comprehensive HIV prevention and PrEP access, which have fallen in the past year [20]. And funding volatility produces supply chain disruptions and worker attrition, with loss of institutional knowledge and community trust [11, 12]. Some of this vulnerability reflects how exceptional management and delivery of HIV programmes have been. For two decades, HIV commodities have moved through dedicated supply chains, supported by dedicated funding and monitored by dedicated reporting systems. That focused approach has, in some cases, permitted better access and outcomes for HIV than other conditions. If unstable support increasingly exposes HIV services to the fragility that unfortunately undermines effective delivery of other health services in many settings impacted by HIV, including frequent stockouts of critical commodities, it could spell tragedy for individual health and rapid loss of population‐wide epidemic control.

This leads to our second qualitative conclusion, which concerns lenacapavir, a twice‐yearly injectable PrEP with near‐perfect clinical trial efficacy [13], that the US government has recently promoted as a centrepiece of its strategy to eliminate VT. With the Global Fund, it initially committed to provide lenacapavir to “2 million people” by 2028 and recently expanded to 3 million [14, 15]. Lenacapavir represents a promising advance for HIV prevention broadly [16], but its impact on VT will depend on the platform through which it is delivered.

Consider some simple arithmetic (Figure 1): UNAIDS estimated about 98,000 new paediatric HIV acquisitions in sub‐Saharan Africa in 2025 [17]. Of these, roughly 24,000 (about 25%) infants were born to mothers who acquired HIV during pregnancy or breastfeeding, the only transmissions that lenacapavir, as PrEP, can address. The remaining 74,000 infants (~75%) were born to women who were already living with HIV before pregnancy and needed ART to prevent transmission.

**FIGURE 1:**
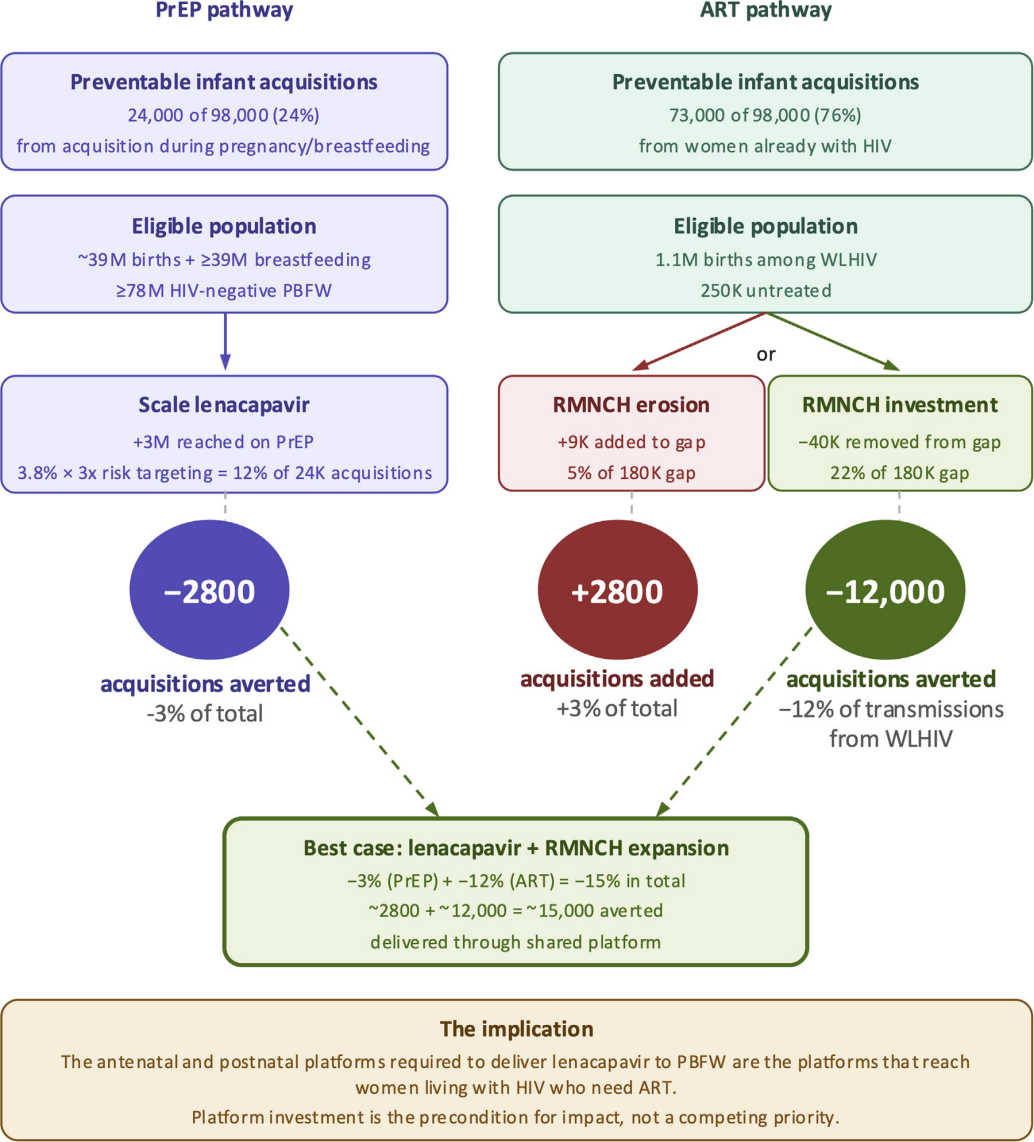
Estimated impact of pre‐exposure prophylaxis and antiretroviral therapy on annual paediatric HIV acquisitions in sub‐Saharan Africa (baseline 98,000). On the left, we calculate a maximum potential impact of lenacapavir delivered as PrEP to pregnant and breastfeeding women under idealized conditions. On the right, we compare this to the potential impact of decreasing or increasing ART initiation and sustained adherence among pregnant and breastfeeding women with HIV. Abbreviations: PrEP, pre‐exposure prophylaxis; ART, antiretroviral therapy; HIV, human immunodeficiency virus; PBFW, pregnant and breastfeeding women; WLHIV, women living with HIV.

Now consider the denominator. PrEP must reach women *without* HIV. In 2024, there were approximately 39 million births among women without HIV in sub‐Saharan Africa [18]. Allocating the entire 3 million‐person commitment exclusively to pregnant and breastfeeding women (unlikely and inefficient) would cover roughly 4% of this population. Even with exceptional targeting to reach those with threefold higher incidence than the general population (see discussion at Ref. [10]), this would avert only 3% of new acquisitions among infants (*n* = 2800) (Figure 1).

This modest direct effect, which relies on strong systems to deliver the medication to those who will benefit most, is why we should think about lenacapavir not as a standalone intervention but as a catalyst for systemic investment in RMNCH services. Of around 1 million women with HIV who gave birth in sub‐Saharan Africa in 2024, an estimated 140,000 did not receive ART, and an additional 40,000 were lost from care [17]. The same family planning, antenatal and postnatal platforms required to identify and reach patients for lenacapavir PrEP are the platforms that could identify and reach women with HIV who need ART. If, for example, a comprehensive RMNCH delivery platform built around lenacapavir also reduced the ART gap by 22%, averting an equivalent percentage of transmissions among women starting pregnancy with HIV as lenacapavir might optimistically achieve among women starting pregnancy without HIV, the impact on VT would exceed fivefold what lenacapavir might achieve on its own.

In sum, we set out to model the impact of discontinued US foreign assistance on VT. We found we could not produce even rough estimates with the available data. What we can say is this: eliminating VT depends on a cascade of services, many of which sit outside the “life‐saving HIV services” still prioritized by US government foreign assistance. Lenacapavir PrEP, for all its promise, will deliver a modest benefit on its own and a substantial one only if it is built on comprehensive RMNCH platforms that also reach women in need of ART.

For the past two decades, the global HIV response has demonstrated what is possible: in several high‐burden countries, ART coverage among pregnant women with HIV has exceeded 95%, showing that elimination of VT is achievable with sufficient resources, systems, data and political will [19]. Our ambition should be to continue this progress and ensure comprehensive delivery of all health services to this same standard.

## Author Contributions

AB, AC and JWI‐E conceptualized the analysis. AB drafted the manuscript. AC, AR, MW, KW and JWI‐E provided critical review and revisions. All authors read and approved the final manuscript.

## Funding

AB reports funding from the National Institute of General Medical Sciences (5R35GM155224). JWI‐E and MW report funding from the National Institute of Allergy and Infectious Diseases of the National Institutes of Health (1R01AI152721) and the UK Medical Research Council (MRC) Centre for Global Infectious Disease Analysis (reference MR/R015600/1), jointly funded by MRC and the UK Foreign, Commonwealth & Development Office (FCDO), under the MRC/FCDO Concordat agreement and is also part of the EDCTP2 programme supported by the EU. AC and KW report funding from the National Institute of Child Health and Human Development of the National Institutes of Health (R37HD079214), and AC reports funding from the James and Audrey Foster MGH Research Scholars.

## Disclaimer

Views expressed are those of the authors and do not necessarily represent those of funders or affiliated institutions, if applicable.

## Conflicts of Interest

The authors declare no conflicts of interest.
